# Circular RNA circitga7 accelerates glioma progression via miR-34a-5p/VEGFA axis

**DOI:** 10.18632/aging.202996

**Published:** 2021-05-07

**Authors:** Ling Qi, Weiyao Wang, Guifang Zhao, Hong Jiang, Yu Zhang, Donghai Zhao, Hong Jin, Hongquan Yu, Haiyang Xu

**Affiliations:** 1 The Sixth Affiliated Hospital of Guangzhou Medical University, Qingyuan People's Hospital, Qingyuan 511518, Guangdong, China; 2 Department of Pathophysiology, Jilin Medical University, Jilin 132013, Jilin, China; 3 Department of Pathology, Jilin Medical University, Jilin 132013, China; 4 Department of Ophthalmology, China-Japan Union Hospital of Jilin University, Changchun 130033, China; 5 Department of Neurovascular, First Hospital of Jilin University, Changchun 130021, China; 6 Department of Oncological Neurosurgery, First Hospital of Jilin University, Changchun 130021, China

**Keywords:** miR-34a-5p, glioma, VEGFA, circular RNA, invasion

## Abstract

Circular RNAs (circRNAs) are a group of noncoding RNAs derived from back-splicing events. CircRNA is reported to be involved in various tumor progressions, including glioma. Although there are a few reports of circular RNAs participating in gliomas, it is still unclear whether circular RNAs regulate the occurrence of gliomas. In our research, we found that the expression of circITGA7 in glioma tissues and glioma cells increased significantly. Knocking down circITGA7 can significantly inhibit the proliferation of glioma cells and reduce cell metastasis. Through analysis and dual-luciferase report assay, we found that circITGA7 acts as a sponge for miR-34a-5p targeting VEGFA in glioma. Our study showed that circITGA7 regulates the proliferation and metastasis of glioma cell lines (SW1783&U373) by regulating the miR-34a-5p/VEGFA pathway. In conclusion, our study revealed a regulatory loop for the circITGA7/miR-34a-5p/VEGFA axis to regulate glioma development.

## INTRODUCTION

Gliomas are classified by WHO into four grades, non-malignant gliomas included grade I and II types, malignant ones consist of grade III and IV types [1], glioblastoma (GBM) is considered as grade IV type gliomas and is often related to poor prognosis [2–4]. Currently, radiotherapy and chemotherapy with temozolomide are still the main therapeutic methods for glioblastomas. However, due to the unsatisfactory therapeutic effect, the expected survival time of these patients is very short [5]. Therefore, making an accurate diagnosis for glioma at the earliest possible stage is very critical for improving treatment efficacy. Although there are new technological instruments that can help confirming the diagnosis of gliomas, such as brain magnetic resonance imaging (MRI) with spectroscopy and perfusion, the high cost hinders large scale application [6]. Advances in molecular biology techniques provide powerful and convenient ways for screening tumors [7]. Therefore, it is of great clinical value to uncover the unknown mechanisms regarding the development of glioma.

Circular RNA (circRNA) was a member of non-coding RNA and possessed a covalently closed consecutive loop. Unlike linear RNA, circRNA can stably reside in the cell for a longer time due to its unique covalent loop structure with neither 5′ to 3′ polarity nor a polyadenylated tail [8]. In recent years, a vast amount of studies has demonstrated that circRNA have important biological functions. For example, Liang et al displayed that circ-ABCB10 promotes breast cancer cell growth [9]. Yang et al demonstrated that circ-ITCH level was down-regulated in bladder cancer cell *in vitro* and *in vivo*. Meanwhile, overexpression of circ-ITCH suppressed bladder cancer cell proliferation and metastasis [10]. Tian et al found that down-regulation of hsa_circ_0003159 expression level had negative links to the extent of remote metastasis and stage of tumor-node-metastasis of gastric cancer (GC) patients [11]. Li et al found that circ-104916 level was lower in GC tissues and cells than adjacent normal tissues. Li et al reported that circITGA7 inhibits colorectal cancer growth and metastasis by modulating the Ras pathway [12]. However, enhanced circ-104916 would greatly retarded GC cell growth *in vitro* [13]. Therefore, analysis the role of circRNA in the occurrence and development of glioma is necessary.

Although some researches have been performed on glioma about circRNAs, the exact mechanism of circRNA for glioma is elusive. Here, we identified and studied a circRNA circITGA7 in glioma. Our study showed that circITGA7 was enhanced in glioma tissues and cells compared to nearby normal ones. Knockdown circITGA7 significantly inhibited cell proliferation and metastasis. Mechanistically, our data revealed that circITGA7 reduced the expression of miR-34a-5p, promoted translation of downstream gene VEGFA, which promoted the growth of glioma.

## RESULTS

### Construction of circITGA7 regulating ceRNA networks

**Figure 1:**
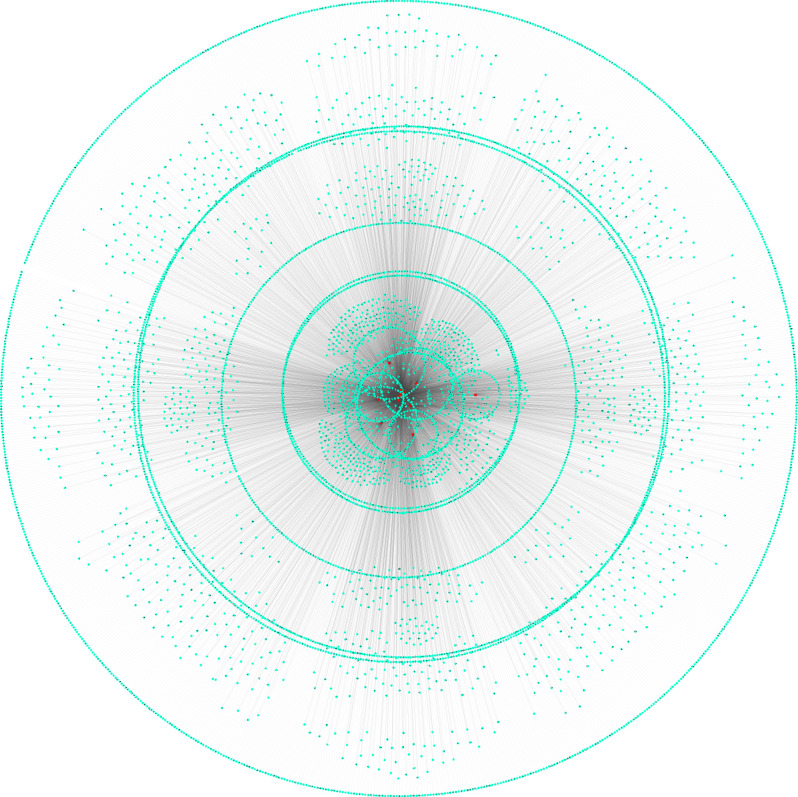
**Construction of circITGA7 regulating ceRNA networks.** Direct targets of circITGA7 targeting miRNAs were predicted, 14 miRNAs and 6068 mRNAs were predicted by regrna2 and Starbase 2.0 database.

Previous studies showed circRNAs could act as miRNA sponges in human cancers [14]. The circITGA7 targeting miRNAs were obtained using RegRNA2.0 database (http://regrna2.mbc.nctu.edu.tw/index.html). Then, the direct targets of circITGA7 targeting miRNAs were predicted using Starbase2.0 database (http://starbase.sysu.edu.cn/). As presented in Figure 1, a total of 14 miRNAs (miR-17-3p, miR-198, miR-34a-5p, miR-191-5p, miR-125a-3p, miR-185-5p, miR-200a-5p, miR-378a-5p, miR-339-5p, miR-432-5p, miR-498, miR-551a, miR-92b-3p) and 6068 mRNAs were identified.

**Figure 2:**
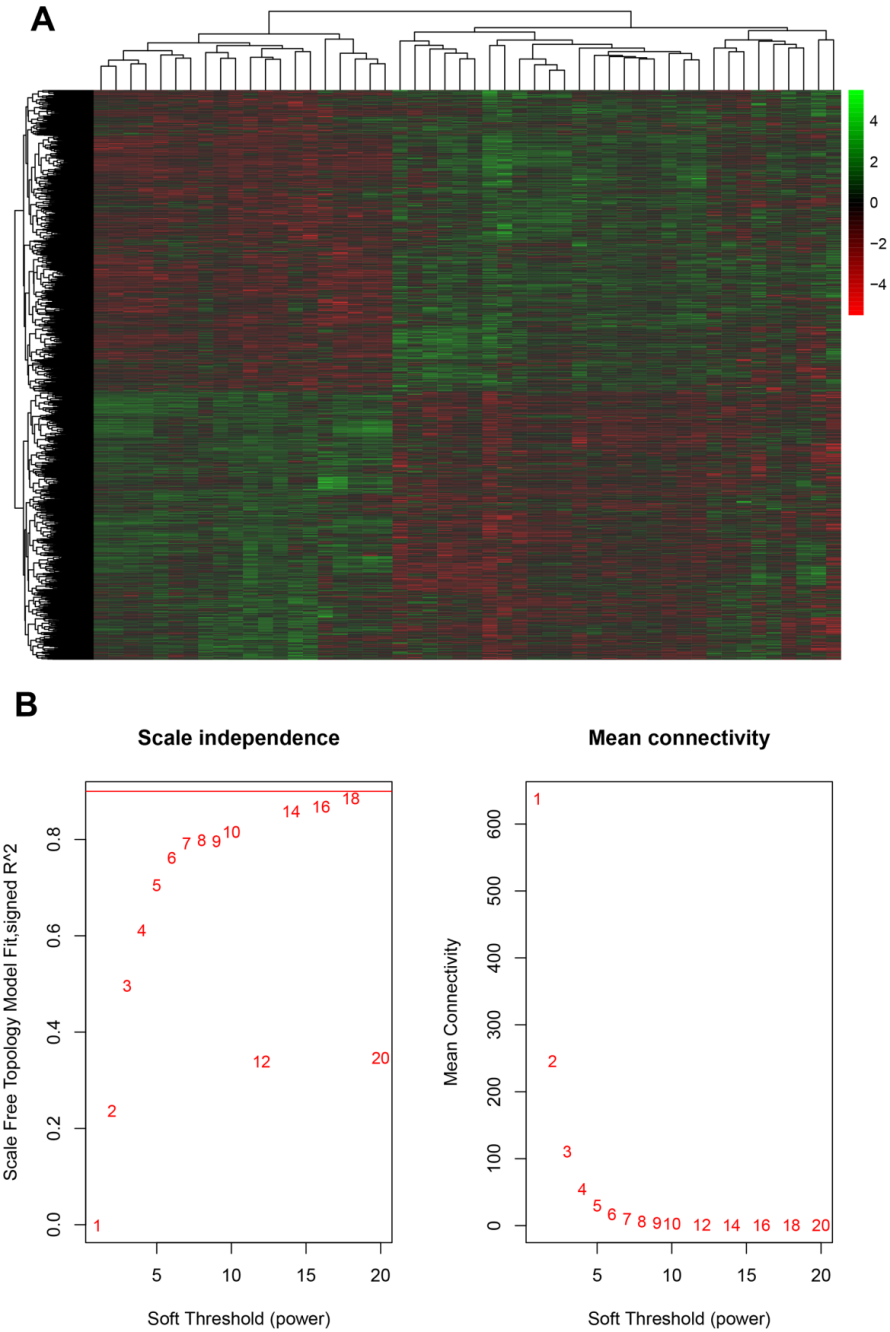
**Evaluation of mRNA and glioma progression.** (**A**) Analysis of the public database GSE43378 identified 1254 down-regulated and 1587 up-regulated potential targets of circITGA7, and (**B**) identified the appropriate power value (soft threshold).

In order to evaluate whether these mRNAs were associated with the progression of gliomas, we analyzed a public database GSE43378, which included 32 GBM samples and 18 LGG samples. A total of 1254 down-regulated and 1587 up-regulated potential targets of circITGA7 were identified in this study (Figure 2).

### Identification key gene modules by WGCNA

**Figure 3:**
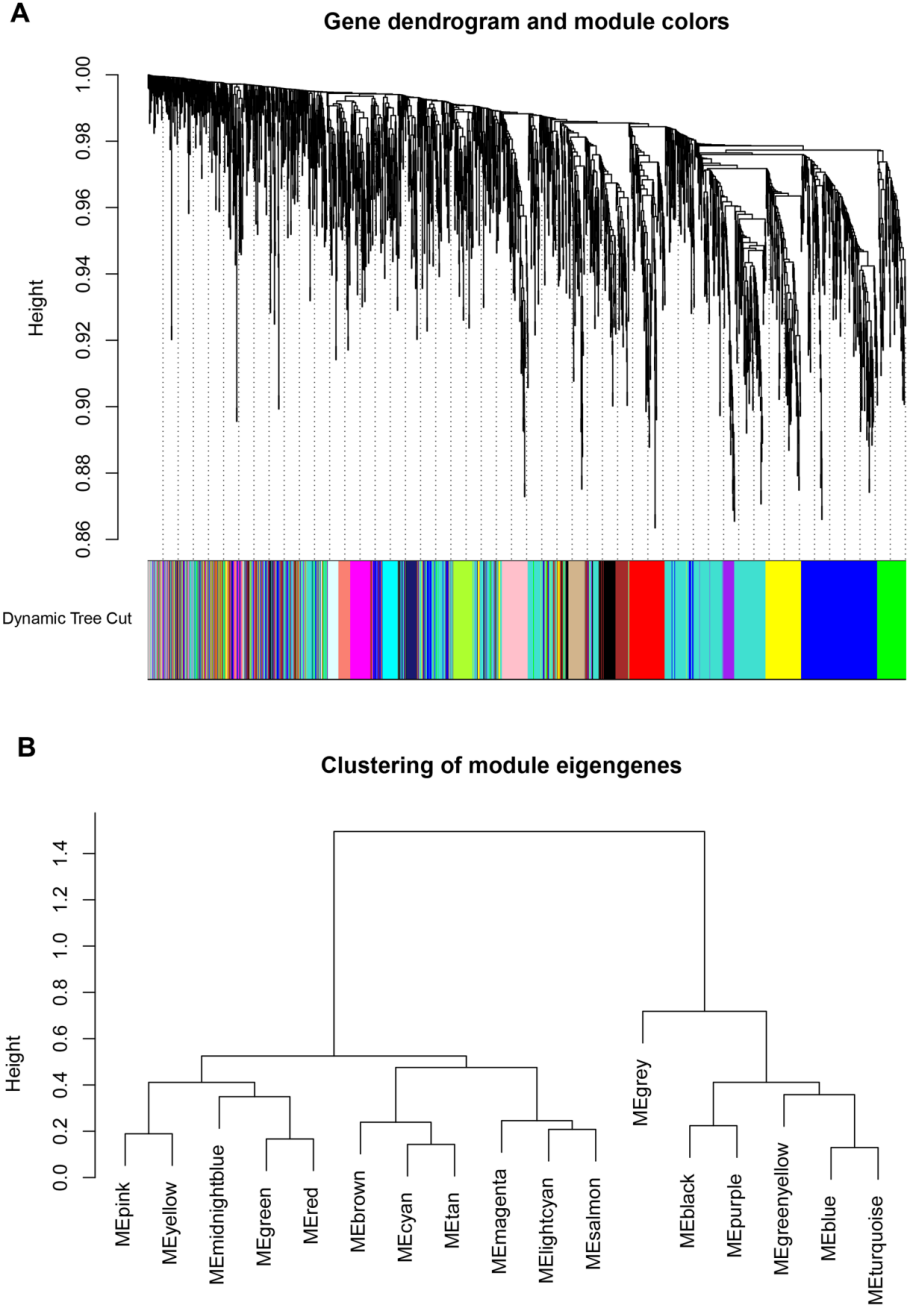
**Identification key gene modules by WGCNA.** (**A**, **B**) Gene cluster dendrogram and module colors. Each color represents the independent module which contains a group of highly correlated genes. A total of 3 modules were identified.

The WGCNA analysis was constructed using R software. The results of the parameter analysis are shown in Figure 2A. After determining the optimal parameter (β=4), the WGCNA algorithm was used to convert the correlation coefficient of a gene pair into the adjacent coefficient. We finally obtained 3 gene modules (Figure 3). A total of 590 genes were included in Module 1, 366 genes were included in Module 2, and 189 genes were included in Module 3.

### Bioinformatics analysis of key targeting modules of circITGA7 in glioma

**Figure 4:**
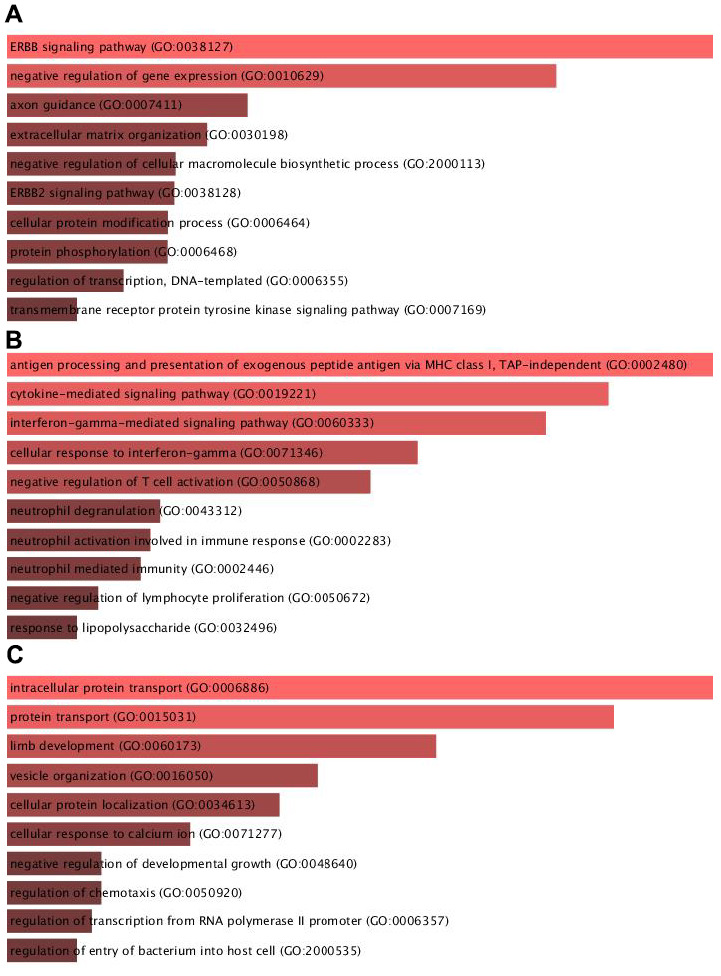
**Bioinformatics analysis of key targeting modules of circITGA7 in glioma.** Bioinformatics analysis of the key targeting module of circITGA7 in glioma was carried out by DAVID system. (**A**) Module 1 is mainly involved in regulating the ERBB signaling (GO: 0038127), (**B**) module 2 is mainly involved in antigen processing and presentation of exogenous peptide antigen via MHC class I, TAP-independent (GO:0002480), and (**C**) module 3 is mainly involved in regulation of intracellular protein transport (GO:0006886).

Then, we applied bioinformatics analysis of key targeting modules of circITGA7 in glioma using DAVID system. Module 1 was involved in regulating ERBB signaling, negative regulation of cellular macromolecule biosynthetic process, ERBB2 signaling, cellular protein modification process, and protein phosphorylation. Module 2 was related to antigen processing and presentation of exogenous peptide antigen via MHC class I, cytokine-mediated signaling, interferon-gamma-mediated signaling, cellular response to interferon-gamma, neutrophil degranulation, neutrophil activation involved in immune response, neutrophil mediated immunity, negative regulation of lymphocyte proliferation. And Module 3 was involved in regulating intracellular protein transport, protein transport, limb development, vesicle organization, cellular protein localization, regulation of chemotaxis, regulation of transcription from RNA polymerase II promoter (Figure 4).

### Identification of key targets of circITGA7 in glioma

**Figure 5:**
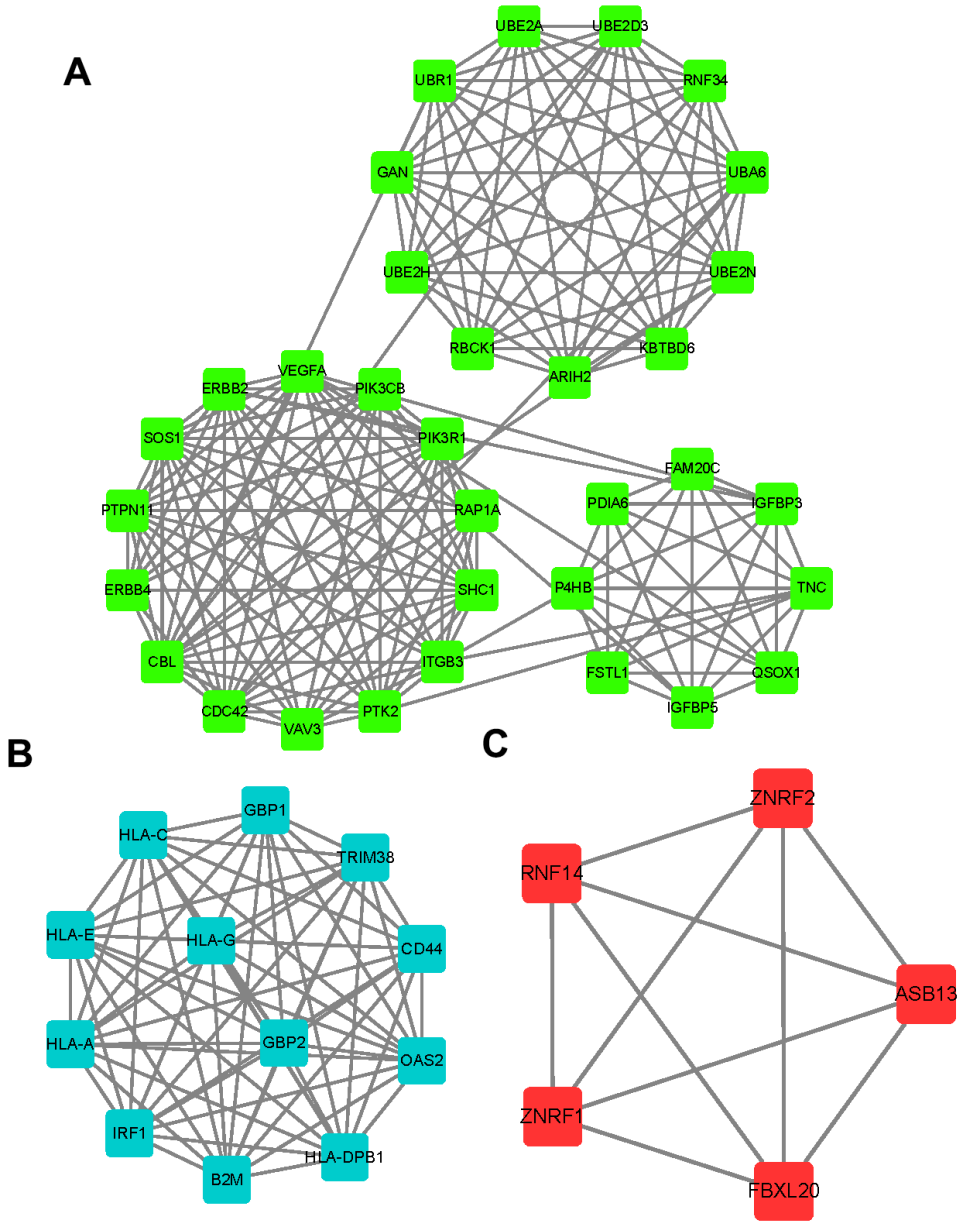
**Identification of key targets of circITGA7 in glioma.** Using a string database, PPI network analysis was used to identify the key targets of circITGA7 in gliomas. 33 key targets were identified in module 1 (**A**), 12 key targets in module 2 (**B**), and 5 key targets in module 3 (**C**).

Then, we applied PPI network analysis to identify key targets of circITGA7 in glioma using String database. As shown in Figure 5, a total of 33 key targets in module 1 were identified, including SHC1, PTK2, CBL, ERBB4, VAV3, CDC42, RNF34, GAN, UBE2D3, UBR1, UBE2A, UBA6, KBTBD6, UBE2H, RBCK1, ARIH2, ERBB2, RAP1A, PIK3CB, PIK3R1, VEGFA, SOS1, PTPN11, P4HB, PDIA6, FAM20C, TNC, IGFBP3, IGFBP5, FSTL1, ITGB3, UBE2N, QSOX1. 12 key targets in module 2 were identified, including HLA-DPB1, HLA-A, B2M, GBP2, IRF1, TRIM38, HLA-G, OAS2, HLA-E, CD44, HLA-C, and GBP1. 5 key targets in module 3 were identified ZNRF2, ZNRF1, RNF14, ASB13, FBXL20.

### Increased circITGA7 level was shown in glioma tissues

**Figure 6:**
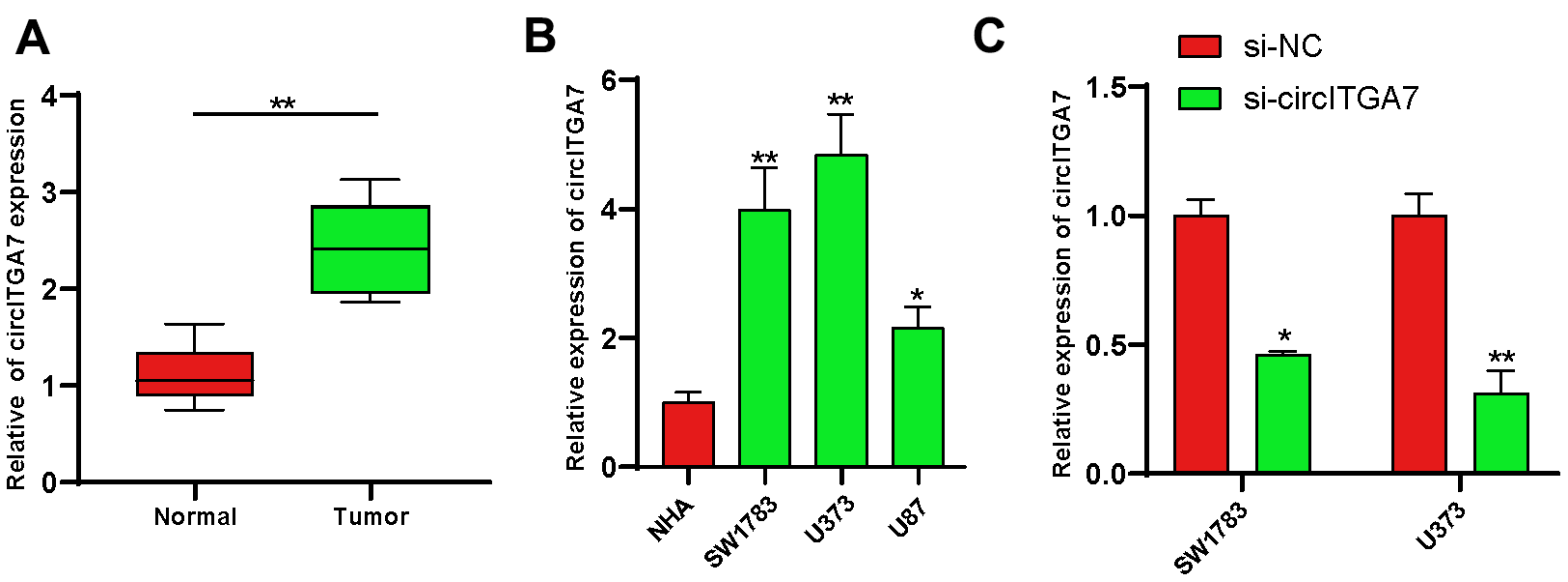
**Increased circITGA7 level was shown in glioma tissues.** (**A**) Relative expression of circITGA7 in 12 pairs of glioma tissues and adjacent normal tissues by qRT-PCR. (**B**) Relative expression of circITGA7 in different glioma cell lines by qRT-PCR. (**C**) Relative expression of circITGA7 in SW1783 and U373 cells transfected with si-circITGA7. *p < 0.05, **p < 0.01.

We firstly evaluated the expression of circITGA7 levels in glioma tissues. For that purpose, 12 pairs of glioma tissues and adjacent normal tissues were subjected to analysis. Our data revealed circITGA7 level was raised in glioma tissues (Figure 6A).

### Evaluation of circITGA7-induced effects on glioma cell proliferation, and metastasis

**Figure 7:**
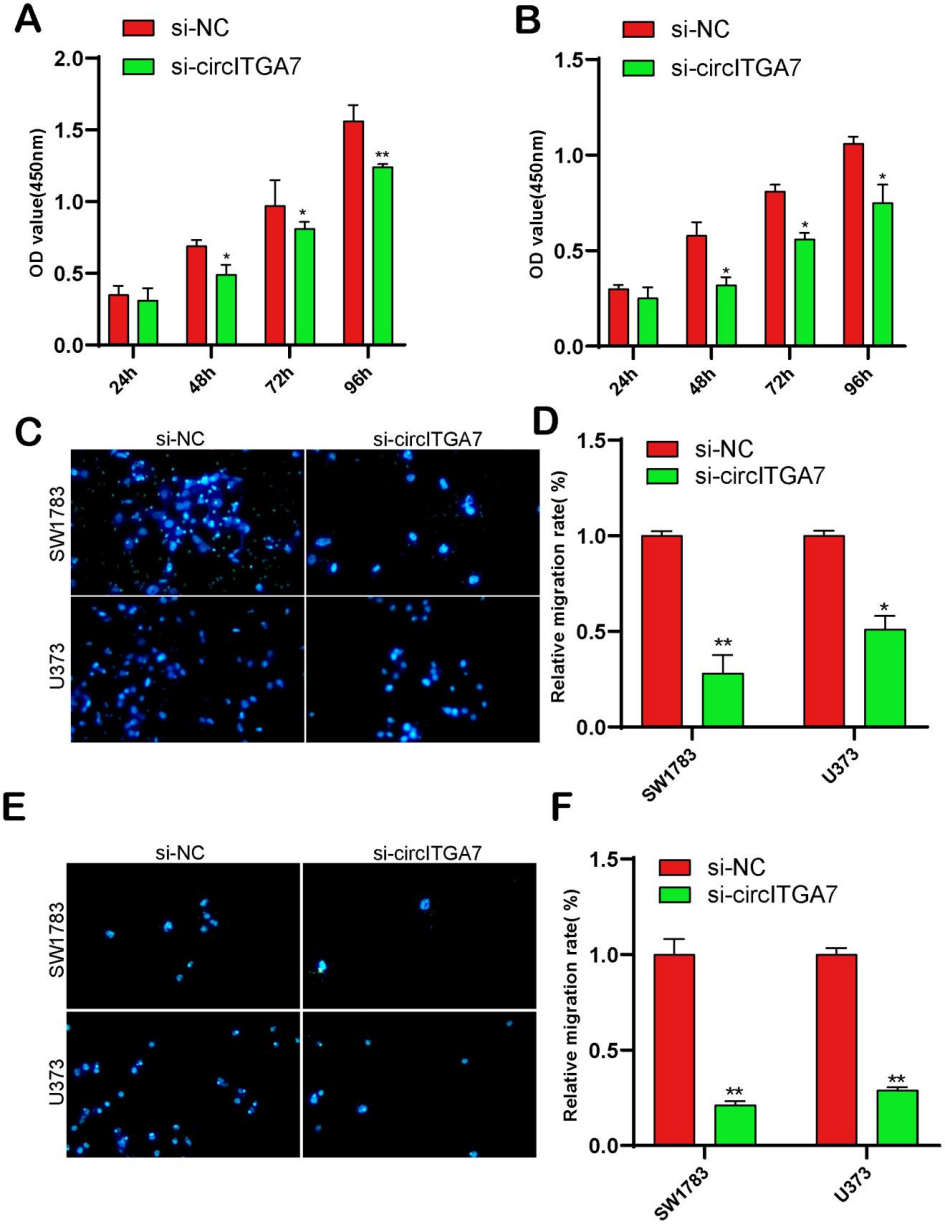
**CircITGA7-induced effects on glioma cell growth, migration, and invasion.** (**A**, **B**) Knockdown of circITGA7 decreased the proliferation in both SW1783 and U373 cells. (**C**–**F**) Cell migration and invasion was determined by transwell assay in SW1783 and U373 cells transfected with si-circITGA7 or NC. *p < 0.05, **p < 0.01.

Here, we determined circITGA7 expression in glioma cell lines to explore the circITGA7-mediated influence on glioma. Our data revealed circITGA7 level was higher in glioma cells compared to that in NHA cells. Of them, SW1783&U373 cells highly expressed circITGA7 (Figure 6B) and were used for the following studies. Figure 6C demonstrated circITGA7 expression levels after knockdown of circITGA7 in SW1783&U373 cell lines. CCK-8 assay shown knockdown circITGA7 marred SW1783&U373 cell proliferation (Figure 7A, 7B). Further data revealed the circITGA7 level was negatively related to metastasis. Transwell demonstrated down-regulation of circITGA7 restrained SW1783&U373 cell migration and invasion (Figure 7C–7F).

### CircITGA7 functioned as a sponge of miR-34a-5p

**Figure 8:**
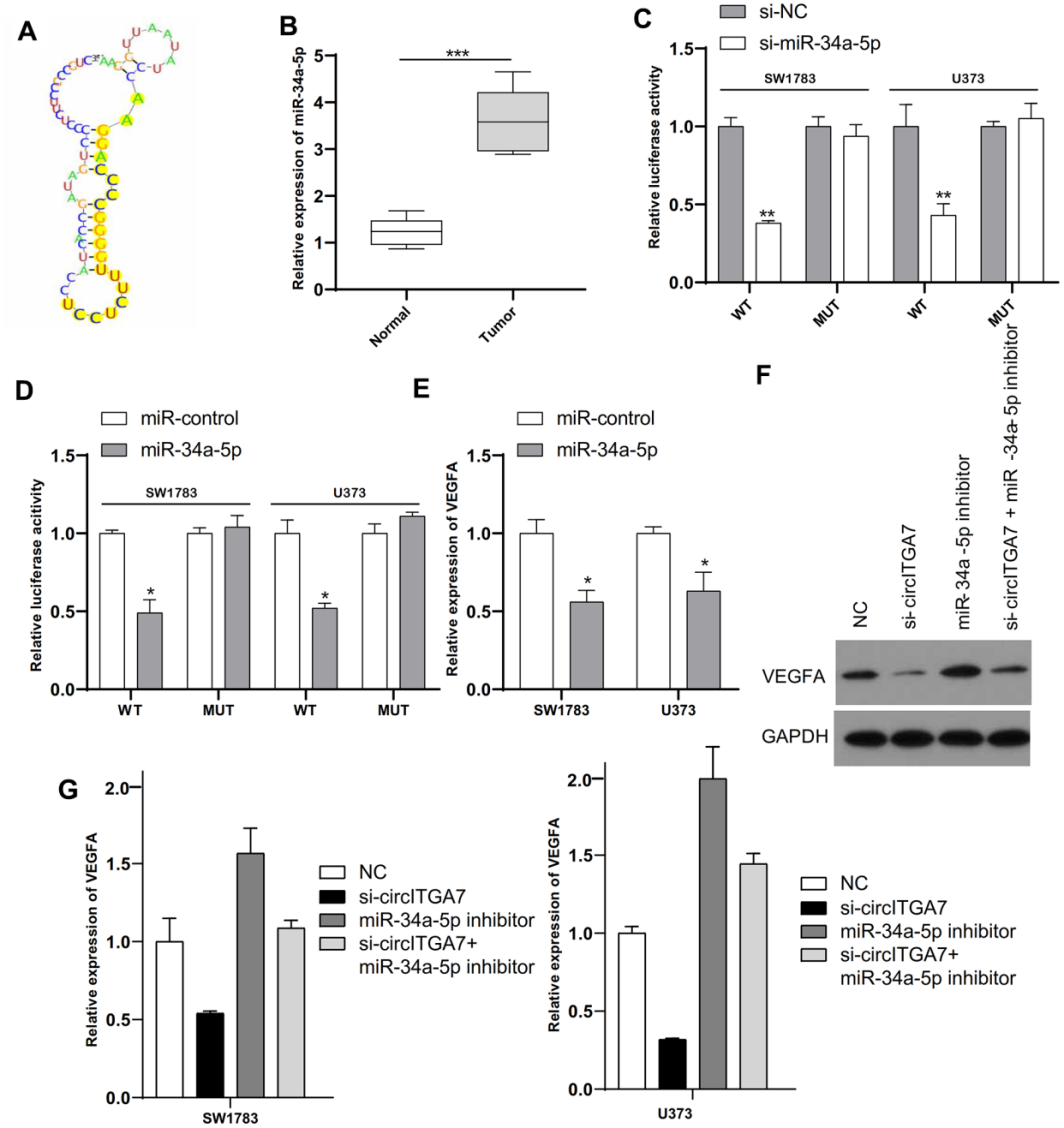
**CircITGA7 functioned as a sponge of miR-34a-5p and VEGFA was a target of miR-34a-5p in glioma.** (**A**) The binding site of miR-34a-5p in circITGA7. (**B**) Relative expression of miR-34a-5p in glioma tissues and paired adjacent normal tissues. (**C**) SW1783 and U373 were transfected with wide-type or mutant circITGA7 reporter plasmid, and the luciferase reporter was performed to confirm the direct target site. (**D**) SW1783 and U373 cells were transfected with wide-type or mutant VEGFA reporter plasmid, and the luciferase reporter was performed to confirm the direct target sites. (**E**) Overexpression of miR-34a-5p inhibited the levels of VEGFA in SW1783 and U373 cells by qRT-PCR. (**F**) Western blot results showed that the level of VEGFA protein in cells transfected with si-circITGA7 decreased, while the level of VEGFA protein in cells transfected with miR-34a-5p inhibitor increased, compared with the control group, miR-34a-5p inhibitor can reduce si-circITGA7 decreases the level of VEGFA protein. GAPDH was as the control. (**G**) The miR-34a-5p inhibitor eliminated the inhibitory effect of circITGA7 knockdown on VEGFA in SW1783 and U373 cells. *p < 0.05, **p < 0.01, ***p<0.001.

Previous researches have shown circular RNAs generally played as sponges of miRNAs through interaction with RISC complex [15]. We predicted probable targets of circITGA7 via miRBase and the CircInteractome. Figure 8A, 8B showed that miR-34a-5p is a potential target of circITGA7 and its expression level in glioma tissue is reduced, which is inversely correlated with the level of circITGA7. Luciferase assay suggested that miR-34a-5p mimic muffled circITGA7-WT-caused effects on SW1783&U373 cells (Figure 8C). Based on the above data, our findings indicated circITGA7 was a sponge for miR-34a-5p and impeded its availability.

### MiR-34a-5p targets VEGFA in gliomas

Emerging data suggested miRNAs functioned through interaction with 3ʹ-UTR of target mRNAs [16]. Therefore, we unearthed targets of miR-34a-5p by the TargetScan and miRanda databases. We selected VEGFA for the study. Luciferase assay indicated miR-34a-5p mimics impeded the activity of WT-VEGFA reporter (Figure 8D). What is more, overexpressed miR-34a-5p obviously inhibited VEGFA level in SW1783&U373 cells (Figure 8E). Furthermore, western blot results showed that VEGFA protein level was decreased in cells transfected with si-circITGA7, and increased in cells transfected with miR-34a-5p inhibitor, and that miR-34a-5p inhibitor attenuated the VEGFA protein level decrease by si-circITGA7 compared with the control group (Figure 8F). Considering our data have displayed miR-34a-5p level could be suppressed by circITGA7, and VEGFA was a target of miR-34a-5p, we further investigated the underlying mechanism in circITGA7-regulated VEGFA expression. Collectively, our data indicated that VEGFA level was increased by circITGA7 via sponging miR-34a-5p (Figure 8G).

### CircITGA7-induced change of glioma cell migration and invasion by regulating miR-34a-5p/VEGFA pathway

**Figure 9:**
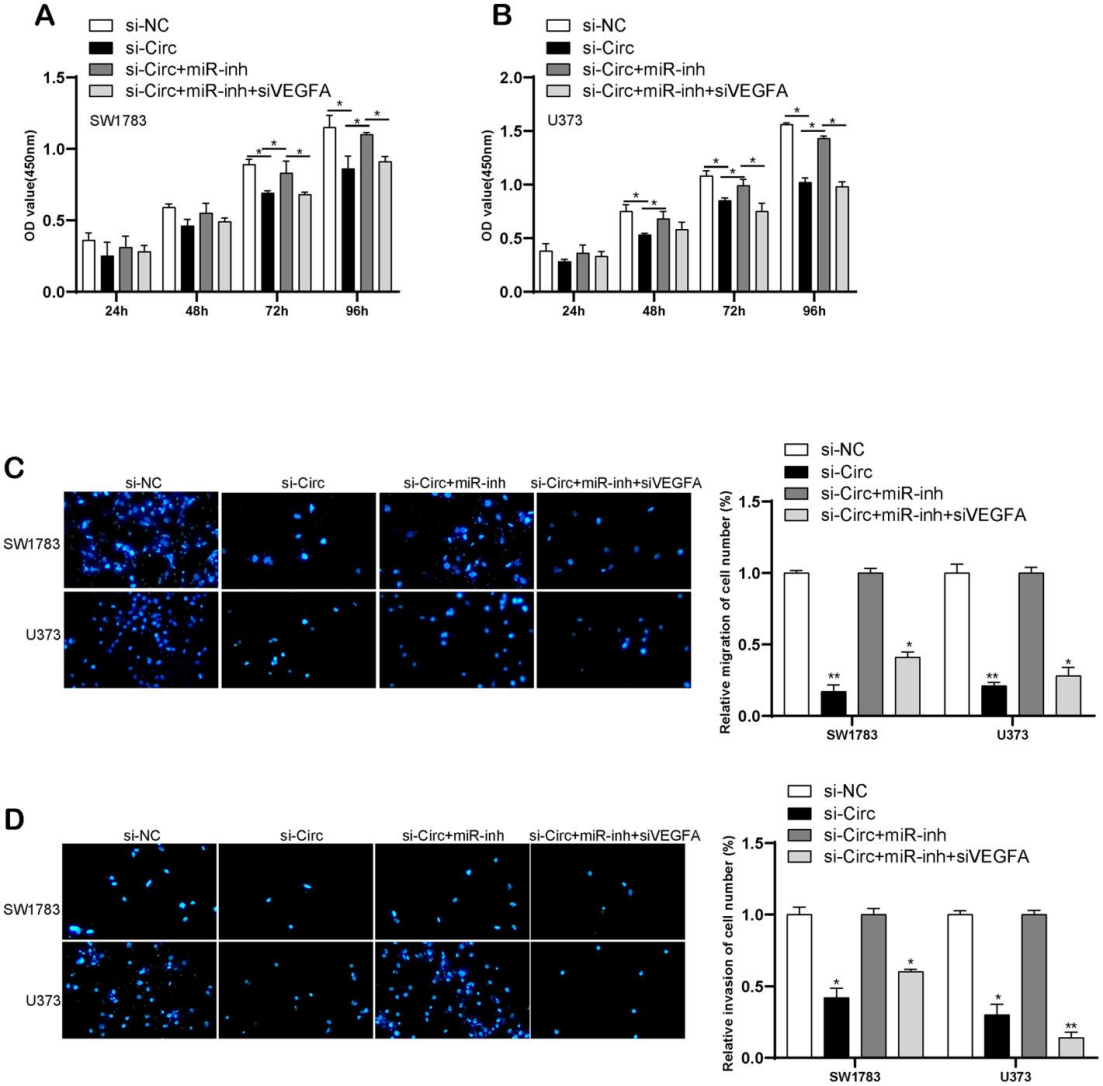
**CircITGA7-induced change of glioma cell metastasis by regulating miR-34a-5p/VEGFA pathway.** (**A**, **B**) CCK-8 assay was used to determine cell growth in SW1783 and U373 transfected with indicative plasmids. (**C**, **D**) Transwell assay was utilized to examine cell migration and invasion in SW1783 and U373 transfected with indicative plasmids. *p < 0.05, **p < 0.01.

We performed the rescue assay to validate circITGA7-induced altered effects on SW1783&U373 cell metastasis after knockdown of miR-34a-5p or VEGFA. CCK-8 and transwell assays revealed downregulated circITGA7 inhibited SW1783&U373 cell growth and metastasis. Nevertheless, suppressed miR-34a-5p induced glioma cell growth, migration and invasion, whereas those effects could be reversed once down-regulation of VEGFA in glioma cells (Figure 9A–9D). Our findings have shown circITGA7 and VEGFA functioned as a promoter in glioma, while miR-34a-5p was regarded as an inhibitor of oncogene. Furthermore, circITGA7 functioned through regulating miR-34a-5p/VEGFA signaling.

## DISCUSSION

The circular RNA remains relatively stable within eukaryotic cells, and has a regulatory role for the life activities of the cells [17]. A large number of clinical reports have found that circRNA functioned importantly in numerous disease processes, like myocardial infarction, osteoarthritis, heart failure, bladder carcinoma, esophageal cancer et al. [18–20]. Previous studies have shown that the circRNA can control miRNA-mRNA signaling networks via up or down-regulation expression level of itself, which involved the human disease process [21]. In the current study, we addressed the expression pattern and roles of circITGA7 in glioma.

With the development of RNA sequencing and synthetic technology, a large number of novel circRNAs were identified. Extensive research efforts have been dedicated to understanding the functions and mechanisms of circRNAs in cancer [22]. Previous studies have shown loss-regulation of circRNA is strongly linked to the processes of many cancer, like basal cell carcinoma, breast cancer, glioma, lung cancer [23–26]. CircRNA has been found extensively participates in the physio pathological processes of tumor. For instance, Xia et al found that knockdown of circ-CBFB remarkably resulted in inhibition of chronic lymphocytic leukemia cell growth, arrest of cell cycle development, and induction of cell apoptosis [27]. Fang et al reported that upregulation of circFAT1(e2) inhibited GC cell proliferation, migration, and invasion via sponging miR-548g and up-regulating target gene RUNX1 level in GC cell cytoplasm [28]. However, the roles of circITGA7 in gliomas remained largely unknown. In this study, we identified 14 miRNAs and 6068 mRNAs as potential targets of circITGA7. Using GSE43378 database, 1254 targets were found to be negatively and 1587 targets were found to be positively correlated to the progression of glioma. Then, we conducted WGCNA analysis to identify key modules and targets of circITGA7. Three key modules and 50 key targets were obtained. Bioinformatics analysis revealed circITGA7 may be involved in regulating ERBB signaling.

Our results have shown that circITGA7 is up-regulated in glioma tissues and cells compared to that in adjacent normal ones. Glioma cell growth, migration and invasion were hindered once decreasing circITGA7 expression. Our finding implies circITGA7 exhibited a primary role in glioma development. CircRNAs often competed for endogenous miRNAs in cancer cells [14]. MiR-34a-5p was chosen in this part by the bioinformatics approach and luciferase assay. We confirmed the association between circITGA7 and miR-34a-5p in glioma cells. The previous study indicated that miR-34a-5p suppressed human cancers by affecting multiple targets. For example, MiR-34a-5p suppressed cell growth and EMT (epithelial-mesenchymal transition) by targeting LEF1 [29]. In gliomas, miR-34a was found to attenuate glioma cells progression and chemoresistance via targeting PD-L1 [30]. Our results indicated that miR-34a-5p interacts with circITGA7. Functional assays also showed miR-34a-5p suppressed gliomas proliferation.

A lot of researches show that miRNAs are involved in cancer initiation and development through regulating targeted mRNAs [31–33]. We identified VEGFA as a direct downstream target gene via bioinformatics prediction. Subsequently, we performed a series of verification experiments to confirm the results of this prediction. As a result, we showed circITGA7 promoted VEGFA expression via sponging miR-34a-5p in glioma. VEGFA is found to be associated with tumor proliferation, migration and invasion. Targeting VEGFA signaling have been reported to be a promising strategy in human cancers [34]. Our data indicated that VEGFA level was increased in glioma tissues and played a carcinogenesis role. Moreover, VEGFA expression was regulated by circITGA7 and miR-34a-5p together in glioma. The silencing of circITGA7 significantly enhanced the expression level of miR-34a-5p and reduced the expression level of VEGFA.

Several limitations should be noted in this study. First, this study was mainly based on bioinformatics analysis. Therefore, the detailed interaction among circITGA7, miR-34a-5p and VEGFA should be further verified at mRNA and protein levels *in vivo* and *in vitro* in the near future. At the same time, we will also establish relevant *in vivo* model to further verify the effects of circITGA7 on gliomas growth and metastasis.

Taken together, this study demonstrated that circITGA7 promoted glioma proliferation via regulating miR-34a-5p/VEGFA axis. Our results provide a potential biomarker of the screening and diagnosis for glioma patients, and a potential target for the treatment of glioma.

## MATERIALS AND METHODS

### Samples

Twelve pairs of glioma and normal samples from the patients who were absent of radiotherapy and chemotherapy before surgery were enrolled from our hospital. All the subjects unanimously consented and signed informed consent documents.

### WGCNA and the identification of modules

The WGCNA R package was used to conducted WGCNA analysis according to previous studies [35].

### Cell culture

SW1783, U373, U87 and NHA were derived from the Type Culture Collection of the Chinese Academy of Sciences (Shanghai, China) and cultured in DMEM medium (Invitrogen, CA, USA) supplemented with 10% fetal bovine serum (Gibco, USA) and 1% streptomycin/penicillin under 37° C incubator containing 5% CO_2_.

### Transfection

Si-circITGA7 was ordered from Gene-Pharma (Shanghai, China). 50nM of mimics were separately transfected into indicated cells using Lipofectamine2000 (Invitrogen) as manual’s protocols. The coding sequence of circITGA7 was inserted into a pcD-ciR construct (Geenseed Biotech, China) and then transfected into SW1783 and U373 cells to establish stably overexpressed cell line and screened by G418.

### CCK-8 assay

CircITGA7, miR-34a-5p and VEGFA with corresponding control were transfected into cells and harvested at an indicated time point, followed by reseeding into 96-well plates as 2×10^3^ cells per well. On the following day, CCK-8 (10 μl/well) was added the absorbance OD 450nm of each well was measured with a microplate reader after incubation for 2 h.

### Transwell assay

The migration and invasion of indicated SW1783 and U373 were detected by transwell chamber (Costar, MA, USA) referred to manuals described.

### qRT-PCR assay

Total RNA was isolated from samples using TRIzol reagent and further reversely transcripted into cRNA, qRT-PCR were then conducted using SYBR Green PCR Kit (Thermo Fisher Scientific).

### Western blot

The total proteins were extracted from cells with RIPA lysis buffer mixed with phenylmethylsulfonyl fluoride (PMSF), protein inhibitors, and phosphatase inhibitors (KeyGEN BioTECH, China). Equal amounts of proteins were separated with 10% SDS-PAGE gel and transferred to polyvinylidene difluoride (PVDF) membranes (Millipore, MA, USA). The membranes were blocked with 5% bovine serum albumin (BSA) for 1.5 h and then incubated with primary antibodies: rabbit polyclonal anti-VEGFA (1:1000, ab46154, Abcam, UK) and rabbit anti-GAPDH (1:10000, ab9485, Abcam, UK). Proteins were then detected by an enhanced chemiluminescence system (ECL) reagent (KeyGEN BioTECH, China) after incubation with secondary antibodies for 1 h at room temperature.

### Luciferase assay

CircITGA7-WT, circITGA7-MUT, and VEGFA-3′UTR-wide-type (WT), VEGFA-3′UTR- mutant-type (MUT) were constructed. MiR-34a-5p binding sites inserted into circITGA7 and VEGFA 3′UTR WT or (MUT were separately cloned into pGL3 luciferase construct (Promega, WI, USA). MiR-34a-5p mimics with luciferase reporter constructs and pRL-TK vector (Promega) were individually transfected into glioma cells. Relative luciferase activity was determined as manufacturers described at 24 h post-transfection.

### Statistical analysis

All the representative data was analyzed by GraphPad Prism 6 and presented as the mean ± SD. Differences existed in two comparison groups and multiple groups were resolved by the Student’s t-test or one-way ANOVA analysis. Overall survival analysis was determined by the Kaplan-Meier curve and a significant difference of each was calculated by the log-rank test. The relation between circITGA7 expression and clinical profile was measured by the Chi-square test. A significant difference was presented as P-value was less than 0.05.

## References

[1] Buck JR, McKinley ET, Fu A, Abel TW, Thompson RC, Chambless L, Watchmaker JM, Harty JP, Cooper MK, Manning HC. Preclinical TSPO Ligand PET to Visualize Human Glioma Xenotransplants: A Preliminary Study. PLoS One. 2015; 10:e0141659. 10.1371/journal.pone.0141659 26517124 PMC4627825

[2] Killela PJ, Pirozzi CJ, Healy P, Reitman ZJ, Lipp E, Rasheed BA, Yang R, Diplas BH, Wang Z, Greer PK, Zhu H, Wang CY, Carpenter AB, et al. Mutations in IDH1, IDH2, and in the TERT promoter define clinically distinct subgroups of adult malignant gliomas. Oncotarget. 2014; 5:1515–25. 10.18632/oncotarget.1765 24722048 PMC4039228

[3] Fitzmaurice C, Abate D, Abbasi N, Abbastabar H, Abd-Allah F, Abdel-Rahman O, Abdelalim A, Abdoli A, Abdollahpour I, Abdulle AS, Abebe ND, Abraha HN, Abu-Raddad LJ, et al, and Global Burden of Disease Cancer Collaboration. Global, regional, and national cancer incidence, mortality, years of life lost, years lived with disability, and disability-adjusted life-years for 29 cancer groups, 1990 to 2017: a systematic analysis for the global burden of disease study. JAMA Oncol. 2019; 5:1749–68. 10.1001/jamaoncol.2019.2996 31560378 PMC6777271

[4] Khani P, Nasri F, Khani Chamani F, Saeidi F, Sadri Nahand J, Tabibkhooei A, Mirzaei H. Genetic and epigenetic contribution to astrocytic gliomas pathogenesis. J Neurochem. 2019; 148:188–203. 10.1111/jnc.14616 30347482

[5] Chamberlain MC. Temozolomide: therapeutic limitations in the treatment of adult high-grade gliomas. Expert Rev Neurother. 2010; 10:1537–44. 10.1586/ern.10.32 20925470

[6] Toyooka M, Kimura H, Uematsu H, Kawamura Y, Takeuchi H, Itoh H. Tissue characterization of glioma by proton magnetic resonance spectroscopy and perfusion-weighted magnetic resonance imaging: glioma grading and histological correlation. Clin Imaging. 2008; 32:251–58. 10.1016/j.clinimag.2007.12.006 18603178

[7] Müllauer L. Milestones in pathology-from histology to molecular biology. Memo. 2017; 10:42–45. 10.1007/s12254-016-0307-z 28367253 PMC5357255

[8] Wang M, Yu F, Wu W, Zhang Y, Chang W, Ponnusamy M, Wang K, Li P. Circular RNAs: a novel type of non-coding RNA and their potential implications in antiviral immunity. Int J Biol Sci. 2017; 13:1497–506. 10.7150/ijbs.22531 29230098 PMC5723916

[9] Liang HF, Zhang XZ, Liu BG, Jia GT, Li WL. Circular RNA circ-ABCB10 promotes breast cancer proliferation and progression through sponging miR-1271. Am J Cancer Res. 2017; 7:1566–76. 28744405 PMC5523036

[10] Yang C, Yuan W, Yang X, Li P, Wang J, Han J, Tao J, Li P, Yang H, Lv Q, Zhang W. Circular RNA circ-ITCH inhibits bladder cancer progression by sponging miR-17/miR-224 and regulating p21, PTEN expression. Mol Cancer. 2018; 17:19. 10.1186/s12943-018-0771-7 29386015 PMC5793418

[11] Tian M, Chen R, Li T, Xiao B. Reduced expression of circRNA hsa_circ_0003159 in gastric cancer and its clinical significance. J Clin Lab Anal. 2018; 32:e22281. 10.1002/jcla.22281 28618205 PMC6817154

[12] Li X, Wang J, Zhang C, Lin C, Zhang J, Zhang W, Zhang W, Lu Y, Zheng L, Li X. Circular RNA circITGA7 inhibits colorectal cancer growth and metastasis by modulating the Ras pathway and upregulating transcription of its host gene ITGA7. J Pathol. 2018; 246:166–79. 10.1002/path.5125 29943828

[13] Li J, Zhen L, Zhang Y, Zhao L, Liu H, Cai D, Chen H, Yu J, Qi X, Li G. Circ-104916 is downregulated in gastric cancer and suppresses migration and invasion of gastric cancer cells. Onco Targets Ther. 2017; 10:3521–29. 10.2147/OTT.S136347 28761361 PMC5522828

[14] Kulcheski FR, Christoff AP, Margis R. Circular RNAs are miRNA sponges and can be used as a new class of biomarker. J Biotechnol. 2016; 238:42–51. 10.1016/j.jbiotec.2016.09.011 27671698

[15] Wang HW, Noland C, Siridechadilok B, Taylor DW, Ma E, Felderer K, Doudna JA, Nogales E. Structural insights into RNA processing by the human RISC-loading complex. Nat Struct Mol Biol. 2009; 16:1148–53. 10.1038/nsmb.1673 19820710 PMC2845538

[16] Rigoutsos I, Furnari F. Gene-expression forum: decoy for microRNAs. Nature. 2010; 465:1016–17. 10.1038/4651016a 20577197

[17] Wang PL, Bao Y, Yee MC, Barrett SP, Hogan GJ, Olsen MN, Dinneny JR, Brown PO, Salzman J. Circular RNA is expressed across the eukaryotic tree of life. PLoS One. 2014; 9:e90859. 10.1371/journal.pone.0090859 24609083 PMC3946582

[18] Lee EC, Elhassan SA, Lim GP, Kok WH, Tan SW, Leong EN, Tan SH, Chan EW, Bhattamisra SK, Rajendran R, Candasamy M. The roles of circular RNAs in human development and diseases. Biomed Pharmacother. 2019; 111:198–208. 10.1016/j.biopha.2018.12.052 30583227

[19] Luo YH, Zhu XZ, Huang KW, Zhang Q, Fan YX, Yan PW, Wen J. Emerging roles of circular RNA hsa_circ_0000064 in the proliferation and metastasis of lung cancer. Biomed Pharmacother. 2017; 96:892–98. 10.1016/j.biopha.2017.12.015 29223555

[20] Shabaninejad Z, Vafadar A, Movahedpour A, Ghasemi Y, Namdar A, Fathizadeh H, Pourhanifeh MH, Savardashtaki A, Mirzaei H. Circular RNAs in cancer: new insights into functions and implications in ovarian cancer. J Ovarian Res. 2019; 12:84. 10.1186/s13048-019-0558-5 31481095 PMC6724287

[21] Zhang F, Zhang R, Zhang X, Wu Y, Li X, Zhang S, Hou W, Ding Y, Tian J, Sun L, Kong X. Comprehensive analysis of circRNA expression pattern and circRNA-miRNA-mRNA network in the pathogenesis of atherosclerosis in rabbits. Aging (Albany NY). 2018; 10:2266–83. 10.18632/aging.101541 30187887 PMC6188486

[22] Qu S, Yang X, Li X, Wang J, Gao Y, Shang R, Sun W, Dou K, Li H. Circular RNA: a new star of noncoding RNAs. Cancer Lett. 2015; 365:141–48. 10.1016/j.canlet.2015.06.003 26052092

[23] Abi A, Farahani N, Molavi G, Gheibi Hayat SM. Circular RNAs: epigenetic regulators in cancerous and noncancerous skin diseases. Cancer Gene Ther. 2020; 27:280–93. 10.1038/s41417-019-0130-x 31477805

[24] Yang Z, Xie L, Han L, Qu X, Yang Y, Zhang Y, He Z, Wang Y, Li J. Circular RNAs: regulators of cancer-related signaling pathways and potential diagnostic biomarkers for human cancers. Theranostics. 2017; 7:3106–17. 10.7150/thno.19016 28839467 PMC5566109

[25] Naeli P, Pourhanifeh MH, Karimzadeh MR, Shabaninejad Z, Movahedpour A, Tarrahimofrad H, Mirzaei HR, Bafrani HH, Savardashtaki A, Mirzaei H, Hamblin MR. Circular RNAs and gastrointestinal cancers: epigenetic regulators with a prognostic and therapeutic role. Crit Rev Oncol Hematol. 2020; 145:102854. 10.1016/j.critrevonc.2019.102854 31877535 PMC6982584

[26] Abbaszadeh-Goudarzi K, Radbakhsh S, Pourhanifeh MH, Khanbabaei H, Davoodvandi A, Fathizadeh H, Sahebkar A, Shahrzad MK, Mirzaei H. Circular RNA and diabetes: epigenetic regulator with diagnostic role. Curr Mol Med. 2020; 20:516–26. 10.2174/1566524020666200129142106 31995005

[27] Xia L, Wu L, Bao J, Li Q, Chen X, Xia H, Xia R. Circular RNA circ-CBFB promotes proliferation and inhibits apoptosis in chronic lymphocytic leukemia through regulating miR-607/FZD3/Wnt/β-catenin pathway. Biochem Biophys Res Commun. 2018; 503:385–90. 10.1016/j.bbrc.2018.06.045 29902450

[28] Fang J, Hong H, Xue X, Zhu X, Jiang L, Qin M, Liang H, Gao L. A novel circular RNA, circFAT1(e2), inhibits gastric cancer progression by targeting miR-548g in the cytoplasm and interacting with YBX1 in the nucleus. Cancer Lett. 2019; 442:222–32. 10.1016/j.canlet.2018.10.040 30419346

[29] Wang X, Zhao Y, Lu Q, Fei X, Lu C, Li C, Chen H. MiR-34a-5p inhibits proliferation, migration, invasion and epithelial-mesenchymal transition in esophageal squamous cell carcinoma by targeting LEF1 and inactivation of the hippo-YAP1/TAZ signaling pathway. J Cancer. 2020; 11:3072–81. 10.7150/jca.39861 32226522 PMC7086260

[30] Ma S, Fu T, Zhao S, Gao M. MicroRNA-34a-5p suppresses tumorigenesis and progression of glioma and potentiates temozolomide-induced cytotoxicity for glioma cells by targeting HMGA2. Eur J Pharmacol. 2019; 852:42–50. 10.1016/j.ejphar.2019.03.005 30851271

[31] Farazi TA, Hoell JI, Morozov P, Tuschl T. MicroRNAs in human cancer. Adv Exp Med Biol. 2013; 774:1–20. 10.1007/978-94-007-5590-1_1 23377965 PMC3704221

[32] Masoudi MS, Mehrabian E, Mirzaei H. MiR-21: a key player in glioblastoma pathogenesis. J Cell Biochem. 2018; 119:1285–90. 10.1002/jcb.26300 28727188

[33] Gu C, Shi X, Huang Z, Chen J, Yang J, Shi J, Pan X. A comprehensive study of construction and analysis of competitive endogenous RNA networks in lung adenocarcinoma. Biochim Biophys Acta Proteins Proteom. 2020; 1868:140444. 10.1016/j.bbapap.2020.140444 32423886

[34] Chen X, Zeng K, Xu M, Liu X, Hu X, Xu T, He B, Pan Y, Sun H, Wang S. P53-induced miR-1249 inhibits tumor growth, metastasis, and angiogenesis by targeting VEGFA and HMGA2. Cell Death Dis. 2019; 10:131. 10.1038/s41419-018-1188-3 30755600 PMC6372610

[35] Jiao M, Li J, Zhang Q, Xu X, Li R, Dong P, Meng C, Li Y, Wang L, Qi W, Kang K, Wang H, Wang T. Identification of four potential biomarkers associated with coronary artery disease in non-diabetic patients by gene co-expression network analysis. Front Genet. 2020; 11:542. 10.3389/fgene.2020.00542 32714363 PMC7344232

